# Effect of mirtazapine versus selective serotonin reuptake inhibitors on benzodiazepine use in patients with major depressive disorder: a pragmatic, multicenter, open-label, randomized, active-controlled, 24-week trial

**DOI:** 10.1186/s12991-016-0115-1

**Published:** 2016-10-19

**Authors:** Tasuku Hashimoto, Akihiro Shiina, Tadashi Hasegawa, Hiroshi Kimura, Yasunori Oda, Tomihisa Niitsu, Masatomo Ishikawa, Masumi Tachibana, Katsumasa Muneoka, Satoshi Matsuki, Michiko Nakazato, Masaomi Iyo

**Affiliations:** 1 Department of Psychiatry, Graduate School of Medicine, Chiba University, 1-8-1 Inohana, Chuo-ku, Chiba, 260-8670 Japan; 2 Department of Psychiatry, Chiba University Hospital, 1-8-1 Inohana, Chuo-ku, Chiba, 260-0856 Japan; 3 Research Center for Child Mental Development, Graduate School of Medicine, Chiba University, 1-8-1 Inohana, Chuo-ku, Chiba, 260-8670 Japan; 4 Fujita Hospital, 3292-Ho Yokaichiba, Sosa-shi, Chiba, 289-2146 Japan; 5 Kimura Hospital, 6-19 Higashihoncho, Chuo-ku, Chiba, 260-0004 Japan; 6 Kisarazu Hospital, 2-3-1 Iwane, Kisarazu-shi, Chiba, 292-0061 Japan; 7 Kokoronokaze Funabashi Clinic, 1-26-2 Motomachi, Funabashi-shi, Chiba, 273-0005 Japan; 8 Kokoronokenko Tsudanuma Clinic, 2-13-13 Maebaranishi, Funabashi-shi, Chiba, 274-0825 Japan; 9 Sodegaura Satsukidai Hospital, 5-21 Nagauraekimae, Sodegaura-shi, 299-0246 Japan

**Keywords:** Depression, Mirtazapine, Benzodiazepines, Brain-derived neurotrophic factor, Serum

## Abstract

**Background:**

This study aimed to evaluate whether selecting mirtazapine as the first choice for current depressive episode instead of selective serotonin reuptake inhibitors (SSRIs) reduces benzodiazepine use in patients with major depressive disorder (MDD). We concurrently examined the relationship between clinical responses and serum mature brain-derived neurotrophic factor (BDNF) and its precursor, proBDNF.

**Methods:**

We conducted an open-label randomized trial in routine psychiatric practice settings. Seventy-seven MDD outpatients were randomly assigned to the mirtazapine or predetermined SSRIs groups, and investigators arbitrarily selected sertraline or paroxetine. The primary outcome was the proportion of benzodiazepine users at weeks 6, 12, and 24 between the groups. We defined patients showing a ≥50 % reduction in Hamilton depression rating scale (HDRS) scores from baseline as responders. Blood samples were collected at baseline, weeks 6, 12, and 24.

**Results:**

Sixty-five patients prescribed benzodiazepines from prescription day 1 were analyzed for the primary outcome. The percentage of benzodiazepine users was significantly lower in the mirtazapine than in the SSRIs group at weeks 6, 12, and 24 (21.4 vs. 81.8 %; 11.1 vs. 85.7 %, both *P* < 0.001; and 12.5 vs. 81.8 %, *P* = 0.0011, respectively). No between-group difference was observed in HDRS score changes. Serum proBDNF levels were significantly decreased (*χ* ^2^ = 8.5, *df* = 3, *P* = 0.036) and serum mature BDNF levels were temporarily significantly decreased (*F* = 3.5, *df* = 2.4, *P* = 0.027) in the responders of both groups at week 24.

**Conclusion:**

This study demonstrated mirtazapine as the first-choice antidepressant for current depressive episodes may reduce benzodiazepine use in patients with MDD.

*Trial registration* UMIN000004144. Registered 2nd September 2010. The date of enrolment of the first participant to the trial was 24th August 2010. This study was retrospectively registered 9 days after the first participant was enrolled

## Background

Benzodiazepines and benzodiazepine-like drugs such as zolpidem and zopiclone are widely prescribed to improve insomnia and anxiety symptoms in combination with antidepressants for the pharmacological treatment of major depressive disorder (MDD) [1–3]. Evidence indicates that using benzodiazepines in conjunction with antidepressants in the first short-term treatment of MDD is effective [4, 5] and useful in preventing patients from dropout [4]. However, long-term use of benzodiazepines should be avoided because they elicit cognitive dysfunction, tolerance, dependence, and increase the risk of dementia in patients with MDD [5, 6], although a recent study has reported negative findings for the relationship between benzodiazepine use and the risk of dementia [7]. Therefore, it is important to establish a strategy for improving depression without using benzodiazepines from an early stage.

Mirtazapine is recognized as one of the first-line antidepressants for the treatment of MDD in addition to other antidepressants including selective serotonin reuptake inhibitors (SSRIs) [8, 9]. Mirtazapine has a unique pharmacological profile with not only α_2_-adrenaline receptor antagonist activity but also histamine H_1_ and serotonin (5-HT)_2A_ receptor antagonism, and it has hypnotic-like effects compared to the SSRIs and other first-line antidepressants [10]. In addition, mirtazapine has 5-HT_2c_ receptor antagonist activity, which is thought to be effective in the treatment of anxiety [11]. Moreover, it has been reported that the onset of clinical antidepressant responses to mirtazapine is faster than the onset with SSRIs [12, 13]. Considering that the actions of mirtazapine include hypnotic-like and fast-acting antidepressant effects, we hypothesized that selecting mirtazapine over other antidepressants including SSRIs as the first choice for a current depressive episode could reduce benzodiazepine use in patients with MDD.

Therefore, the primary purpose of this study was to determine whether treatment of current depressive episodes with mirtazapine could reduce the use of benzodiazepine in patients with MDD more than the representative SSRIs, sertraline and paroxetine could. Furthermore, the secondary purpose of this study was to compare the efficacy and safety of these three antidepressants in patients with MDD.

Accumulating preclinical and clinical studies have suggested that the brain-derived neurotrophic factor (BDNF) plays an important role in the pathophysiology of MDD and serum levels of BDNF may have the relationship with clinical responses to treatments for depression [14]. Moreover, recent studies have shown that serum levels of mature BDNF and proBDNF, which is a precursor form of mature BDNF, are successfully measured separately [15–17]. Furthermore, mature BDNF and proBDNF are reported to play different roles in neurophysiological functions via the tropomyosin receptor kinase B (TrkB) and p75 neurotrophin receptors, respectively [14, 18, 19]. Meta-analysis studies have shown that antidepressant treatments influence serum levels of BDNF in patients with MDD [20, 21]. However, the effects of antidepressant treatments on serum levels of mature BDNF and proBDNF in patients who are depressed are not well known. Therefore, we also determined whether serum levels of mature BDNF and proBDNF could be potential biomarkers of clinical responses to antidepressant treatments in patients with MDD.

## Methods

### Study design and participants

We conducted an open-label, randomized, and active-controlled 24-week trial in outpatients with current depressive episodes in routine psychiatric practice settings. The study participants were recruited from 13 sites in Japan, and the study was conducted from September 2010 to March 2014. This study was approved by the Institutional Review Boards and Ethics Committees of all the participating institutes and was performed in accordance with the ethical standards of the Helsinki Declaration of 1975, as revised in 2013. The trial was registered with the Clinical Trials Registry of the University Hospital Medical Information Network (UMIN, Tokyo, Japan, registration number UMIN000004144). All subjects provided written informed consent for their participation in the study after the procedure had been fully explained to them.

The inclusion criteria for prospective participants were: (1) age 20–75 years; (2) diagnosed according to the *Diagnostic and Statistical Manual of Mental Disorders, 4th Edition, Text Revision* criteria for MDD; (3) a ≥12 total score on the 17-item Hamilton depression rating scale (HDRS) [22]; (4) considered to require antidepressant treatment based on the judgment of the consulting psychiatrist. The exclusion criteria for participants were the following: (1) previous history of the use of mirtazapine or both sertraline and paroxetine; (2) pregnant or breastfeeding; (3) at significant risk for suicide; (4) diagnosed with a primary condition including dementia as well as bipolar, obsessive–compulsive, or eating disorders, schizophrenia, or alcohol or substance dependence except for tobacco dependence; (5) experiencing any medical conditions judged to render the patient ineligible to participate in the study.

### Procedures

The participants in this study were treatment-seeking outpatients who personally visited each investigating hospital or clinic to consult about their current depressive symptoms. The participants were provided with the full details of the study modality and were informed that they were responsible for the usual consultation and medicine fees because the study was conducted in the routine psychiatric practice setting. The participants were randomly assigned to the mirtazapine or SSRIs groups in a 1:2 ratio. The computerized randomization program provided by EPS Associates Co., Ltd. (Tokyo, Japan) had a minimization algorithm with two prognostic factors, sex and sleep-related scores of the HDRS (i.e., low 0–3 or high 4–6). The investigators overseeing the SSRIs groups were free to choose either sertraline or paroxetine. If the participant had been taking other antidepressants before participating in this study, the drugs were tapered off during the first 4 weeks. The titration and tapering of the dosage of the investigational antidepressants were flexible and based on the clinical judgment of each investigator throughout the study.

Furthermore, each investigator prescribed benzodiazepines or benzodiazepine-like drugs such as zolpidem and zopiclone for insomnia or anxiety symptoms from the first day of the study after providing a sufficient explanation of the risks involved including dependence and sedation. In principle, the investigators were to prescribe the designated drugs of benzodiazepines for insomnia and anxiety symptoms of the participants. At the same time, they were also free to prescribe other benzodiazepines, zolpidem or zopiclone other than the designated benzodiazepines on the basis of the clinical judgement of each investigator-in-charge. In addition, the participants were provided with directions on how to administer the benzodiazepines according to the drug prescribing information and the original study instructions. Alternatively, the investigators were also allowed to avoid prescribing benzodiazepines when the patients did not wish to take them. The patients were directed to take the benzodiazepines when needed, similar to the pill-in-the-pocket approach according to each patient’s judgment and not on a fixed schedule. The participants were required to maintain a daily record of taking the medication using specific notebooks, which were copied at every visit to check their compliance with the medication use and the use of the benzodiazepines. The patients were not informed that taking the benzodiazepines was one of the clinical outcomes of the study. Furthermore, they were provided with the usual medical consultation but were not treated with the specific psychotherapy for the purpose of reducing benzodiazepine use.

Blood samples were collected between 10:00 a.m. and 4:00 p.m. at baseline and weeks 6, 12, and 24 to measure the serum mature BDNF and proBDNF levels. The serum samples were rapidly delivered to the Department of Psychiatry, Chiba University Graduate School of Medicine in anticoagulant tubes at 4 °C and stored at −80 °C until analyzed.

### Measurements of serum mature and precursor proBDNF levels

The mature BDNF and precursor proBDNF levels were measured using a human proBDNF enzyme-linked immunosorbent assay (ELISA) kit (Biosensis, Thebarton, SA, Australia) and the human mature BDNF ELISA Kit (Aviscera Bioscience, Santa Clara, CA, USA). All experiments were performed in duplicate according to the manufacturer’s instructions. The optical density of the resulting reaction solutions in each well was measured using an automated microplate reader (Emax, Molecular Devices, Sunnyvale, CA, USA).

### Assessments of clinical outcomes

The primary outcome of this study was the proportion of patients using benzodiazepines, denoted as “benzodiazepine users”, at weeks 6, 12, and 24, which was compared for the two (mirtazapine and SSRIs) or three (mirtazapine, sertraline, and paroxetine) investigational groups. The benzodiazepine users and non-users were defined as patients who took benzodiazepine drugs once or more during the 1-week period prior to each assessment points (6, 12 and 24 weeks) or did not, respectively. Based on the frequencies of benzodiazepine use, the participants were distinguished into non-use, 1–6 days per week usage, and everyday usage and benzodiazepine users were defined as those in the 1–6 days per week usage or daily usage categories. To clarify the effect of each antidepressant on the use of benzodiazepines, we determined the number of patients in each group who were prescribed benzodiazepines from the first prescription day of the study and compared the proportion of benzodiazepine users between the groups. Therefore, the patients who did not want benzodiazepine prescriptions on the first day of the study were excluded from the primary outcome assessment.

The secondary outcomes were the efficacy and safety assessments of each antidepressant treatment, which were compared between the groups of patients prescribed benzodiazepines on the first day, and between the groups regardless of benzodiazepine prescription using an intent-to-treat analysis. To assess the severity of depressive symptoms, we used the HDRS and defined patients showing a ≥50 % reduction in HDRS scores from baseline to assessment day as responders, and those who did not as non-responders. We also assessed the self-reported inventory of depression using the Zung self-rating depression scale (SDS) questionnaire [23]. To assess the severity of sleep disturbances, we used the Athens insomnia scale (AIS) [24] and also administered the clinical global impressions-severity (CGI-S) scale [25]. The HDRS, SDS, AIS, and CGI-S scores were measured at baseline and weeks 1, 2, 6, 12, and 24. For the safety assessments, we collected information on all the adverse events (AEs) observed during this study, which were defined as serious AEs such as those leading to death, life-threatening conditions, hospitalizations, or persistent disability.

### Assessments of relationship between clinical responses and serum BDNF levels

To explore the clinical applicability of serum mature BDNF and proBDNF measurements as biomarkers in depression treatment, we specifically examined the relationship between the clinical responses to antidepressant treatments and serum BDNF levels in both antidepressant groups using the following two approaches. One approach involved examining whether the measured baseline serum levels of mature BDNF and proBDNF would be adequate predictors of clinical responses to antidepressant treatments during the acute phase (e.g., 6–8 weeks) of depression treatment. Specifically, we examined the baseline levels of serum mature BDNF and proBDNF between responders and non-responders who were assessed at week 6. The other strategy was to evaluate the long-term effectiveness of antidepressant treatments by examining the associated changes in serum levels of mature BDNF and proBDNF in responders who achieved clinical responses by the final assessment day, week 24. Moreover, we also examined the ratio of the levels of mature BDNF and proBDNF according to a previous study [17].

### Statistical analyses

The analyses of the primary outcome were performed in proportions of the benzodiazepine users at weeks 6, 12, and 24, between the groups of patients who were prescribed benzodiazepines from the first study day using a two-tailed Chi-square test or the Fisher’s exact test.

The analyses of the efficacy outcomes were conducted on an intent-to-treat basis, and using a linear mixed-effects model for repeated measures (MMRM) with treatment group, week, and treatment group-by-week interaction as fixed effects and subject as a random effect. The Bonferroni adjustment was used for the multiple comparisons. The safety analyses were performed for the three groups of patients who took at least one dose of the prescribed antidepressant.

We used parametric tests to analyze the data of the serum mature BDNF levels while non-parametric tests were used for the serum proBDNF levels and the ratio of serum mature BDNF and proBDNF levels because these data did not follow a normal distribution although that of the mature BDNF did. We conducted an independent *t* test or the Mann–Whitney *U* test to compare the baseline levels of BDNF between the responders and non-responders. We used a repeated analysis of variance (ANOVA) for the serum mature BDNF levels while the Friedman’s test was used for the proBDNF levels and the ratio of both proteins to examine the long-term effects of antidepressant treatments on continuous changes in serum BDNF levels.

A *P* < 0.05 was considered statistically significant in all analyses, which were conducted using the statistical package for the social sciences (SPSS) version 23.0 (IBM, NY, US).

We expected the proportions of benzodiazepine users to be 30.0 and 60.0 % in mirtazapine and SSRIs groups, respectively, according to a previous study [1] with an alpha error and power of 5.0 and 80.0 %, respectively. The total sample size of 120 participants was estimated with a consideration of a 20.0 % withdrawal. We allowed this study to be completed ahead of schedule when the result of the primary outcome was obviously confirmed by an interim analysis that was used to detect the difference in the proportions of benzodiazepine users at week 6 between the groups, which showed that the analysis achieved a *P* < 0.001 in the Chi-square test.

## Results

### Participants and clinical course outline

**Fig. 1:**
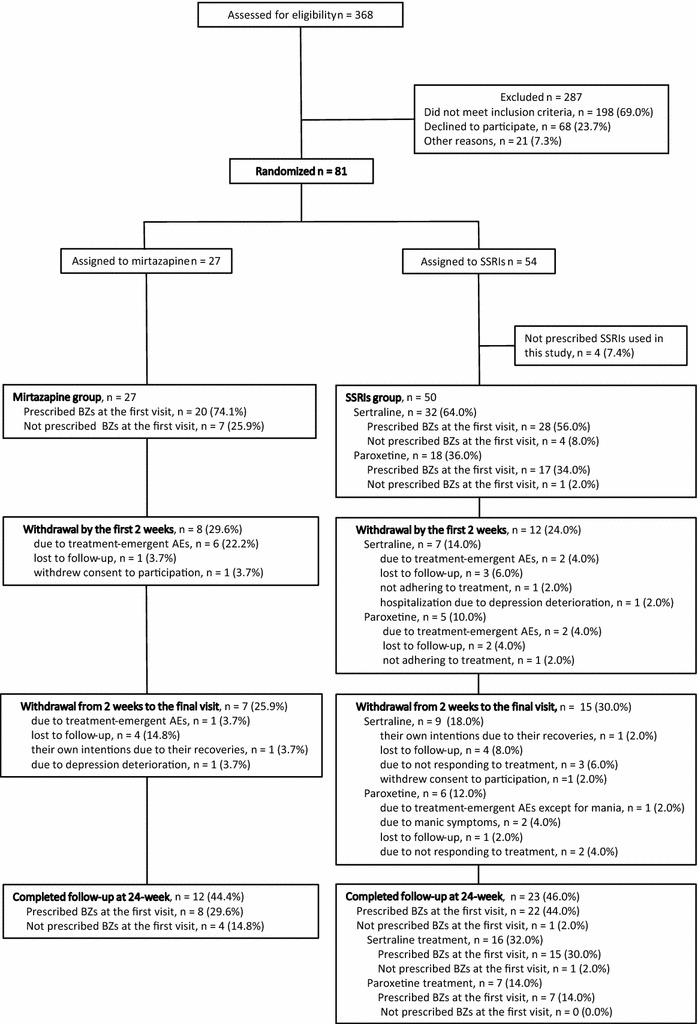
Study flowchart. *AE* adverse event, *BZ* benzodiazepine, *SSRI* selective serotonin reuptake inhibitor

Of the 368 patients screened, 81 were enrolled in this study (Fig. 1). We perform an interim analysis of the data of 77 participants (Table 1) who were ready to be assessed by week 6 and subsequently terminated participant recruitment. The termination was instituted because we confirmed that the primary outcome results of the analysis had achieved a *P* < 0.001, and the proportions of benzodiazepine users in the mirtazapine and SSRIs groups were clearly distinct from each other. This indicated that we required a lower sample size than we originally expected.

**Table 1:** Patient characteristics at baseline

Variable	Mirtazapine (*n* = 27)	SSRIs (*n* = 50)	*P*	Sertraline (*n* = 32)	Paroxetine (*n* = 18)	*P*
Male patients, *n* (%)	18 (66.7)	32 (64.0)	ns^c^	20 (62.5)	12 (66.7)	ns^c^
Age, mean (SD), years	38.9 (10.5)	40.4 (13.8)	ns^a^	39.7 (13.3)	41.7 (14.9)	ns^b^
Age at onset, mean (SD), years	38.1 (10.5)	39.3 (13.1)	ns^a^	39.0 (12.5)	39.8 (14.5)	ns^b^
Duration of illness, median [quartiles], week	30.0 [8.0–104.0]	20.0 [12.0–71.0]	ns^a^	20.0 [8.0–58.5]	19.0 [12.0–117.0]	ns^b^
Duration of current episode, median [quartiles], week	12.0 [7.0–40.0]	12.0 [8.0–34.0]	ns^a^	14.0 [7.3–44.0]	12.0 [12.0–21.0]	ns^b^
Depressive episodes			ns^c^			ns^c^
Single, *n* (%)	22 (81.5)	42 (84.0)		28 (87.5)	14 (77.8)	
Recurrent, *n* (%)^d^	5 (18.5)	8 (16.0)		4 (12.5)	4 (22.2)	
Past history of using any psychiatric services, *n* (%)	4 (14.8)	15 (30.0)	ns^c^	11 (34.4)	4 (22.2)	ns^c^
Past history of any psychotropic medication, *n* (%)	11 (40.7)	18 (36.0)	ns^c^	14 (43.8)	4 (22.2)	ns^c^
Treatments of the current episode, *n* (%)	4 (14.8)	5 (10.0)	ns^c^	5 (15.6)	0 (0.0)	ns^c^
Antidepressant treatment of the current episode, *n* (%)	3 (11.1)	3 (6.0)	ns^c^	3 (9.4)	0 (0.0)	ns^c^
Benzodiazepine treatment of the current episode, *n* (%)	1 (3.7)	1 (2.0)	ns^c^	1 (3.1)	0 (0.0)	ns^c^
HDRS, mean (SD)	23.0 (5.2)	23.1 (6.1)	ns^a^	23.2 (6.2)	22.9 (6.0)	ns^b^
SDS, mean (SD)	55.9 (5.4)	57.9 (7.8)	ns^a^	57.9 (7.4)	57.9 (7.1)	ns^b^
AIS, mean (SD)	11.2 (3.7)	12.9 (4.4)	ns^a^	12.8 (4.3)	13.1 (4.7)	ns^b^
CGI-S, median [quartiles]	4.0 [4.0–5.0]	4.0 [4.0–5.0]	ns^a^	4.5 [4.0–5.0]	4.0 [4.0–5.0]	ns^b^

*SSRI* selective serotonin reuptake inhibitor, *SD* standard variation, *HDRS* 17-item Hamilton depression rating scale, *SDS* Zung self-rating depression scale, *AIS* Athens insomnia scale, *CGI*-*S* clinical global impressions-severity

^a^Unpaired *t* test or Mann–Whitney *U* test

^b^One-way analysis of variance (ANOVA) or Kruskal–Wallis test

^c^Chi-square test or Fisher exact test

^d^Maximum number of recurrent episodes is two

Of the 18 patients assigned to receive paroxetine, ten and eight were prescribed the standard and controlled-release (CR) tablets, respectively. The daily mean peak doses of the antidepressants in this study were 27. 2 ± 11.8, 73.4 ± 28.4, 24.0 ± 8.0, and 37.5 ± 10.8 mg in the mirtazapine and sertraline groups as well as paroxetine standard and paroxetine CR subgroups, respectively. The dose ranges of the mirtazapine, sertraline, paroxetine standard, and paroxetine CR antidepressants were as follows: 15.0–45.0, 25–100, 10–40, and 25–50 mg, respectively. Table 2 shows the breakdown of benzodiazepines prescribed to the 65 patients who were prescribed them from prescription day 1.

**Table 2:** Breakdown of prescribed benzodiazepines

Benzodiazepines	Mirtazapine (*n* = 20)^a^	SSRIs (*n* = 45)^a^	Sertraline (*n* = 28)^a^	Paroxetine (*n* = 17)^a^
As hypnotics				
Brotizolam	12	22	9	13
Estazolam	1	0	0	0
Flunitrazepam	0	5	4	1
Nitrazepam	1	1	1	0
Rilmazafone	0	1	1	0
Triazolam	0	4	3	1
Zopiclone	0	1	1	0
Zolpidem	1	0	0	0
As anxiolytics				
Alprazolam	2	7	1	6
Bromazepam	0	5	5	0
Etizolam	2	5	5	0
Clotiazepam	0	2	2	0
Lorazepam	7	16	9	7

^a^Patients prescribed benzodiazepines at baseline (day 1) were only counted in numbers in this table

### Primary outcome: group proportions of benzodiazepine users

**Fig. 2:**
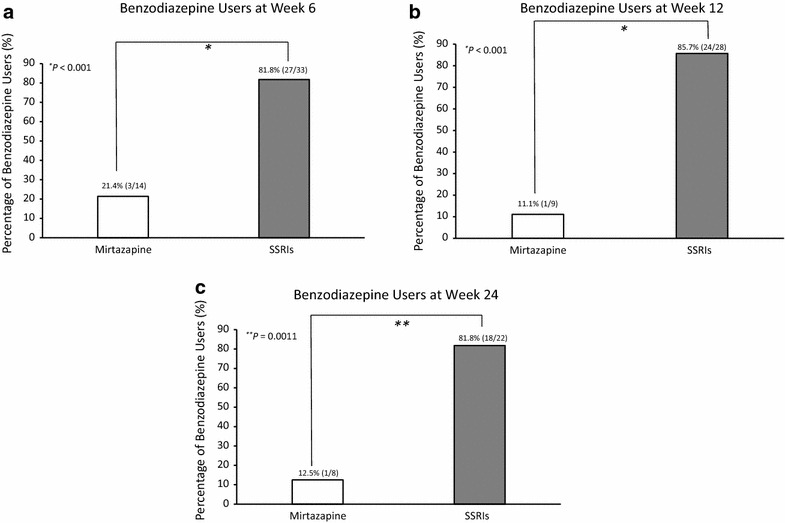
Proportions of benzodiazepine users in mirtazapine and selective serotonin reuptake inhibitors (SSRIs) groups. *Numbers in parentheses* above *bars* are actual numbers of benzodiazepine users and group participants assessed each week. Benzodiazepine users are defined as patients who took benzodiazepine drugs once or more during the 1-week period prior to each assessment point (weeks 6, 12, and 24). Patients were prescribed benzodiazepines from study day 1. *P* values are based on analyses of Chi-square (**a**) and Fisher’s exact test (**b**, **c**). *SSRI* selective serotonin reuptake inhibitor

**Fig. 3:**
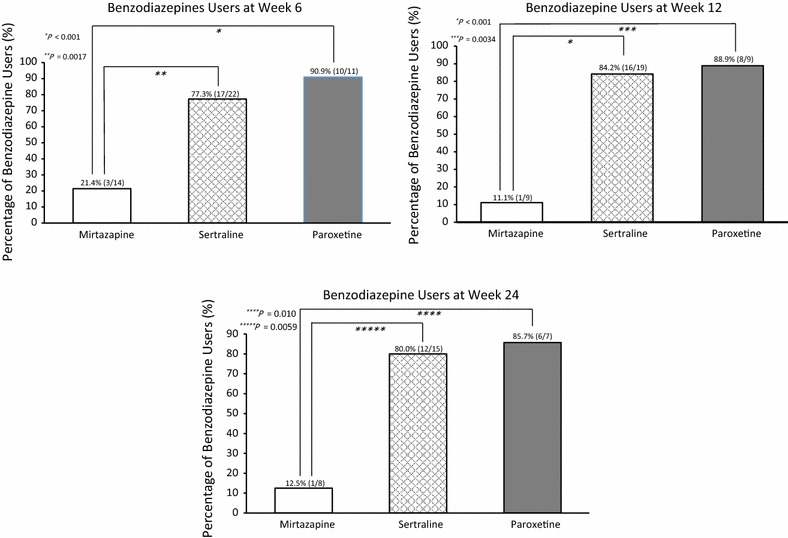
Proportions of benzodiazepine users in three antidepressants groups. *Numbers in parentheses* above *bars* are actual number of benzodiazepine users and group participants assessed each week. Benzodiazepine users are defined as patients who took benzodiazepine drugs once or more during the 1-week period prior to each assessment point (weeks 6, 12, and 24). Patients were prescribed benzodiazepines from study day 1. We analyzed differences in the proportions in two groups using Chi-square test at week 6 (**a**) and Fisher’s exact test at week 12 and 24 (**b**, **c**), after the analyses were conducted among the three groups using Chi-square test at week 6 and the Fisher’s exact test at week 12 and 24. *P* values are based on analyses of Chi-square (**a**) and the Fisher exact test (**b**, **c**)

Table 3 shows the frequencies of the benzodiazepine users for the groups at each assessment point. As shown in Fig. 2, the percentage of benzodiazepine users at week 6 in the mirtazapine group (21.4 %) was significantly lower than that in the SSRIs group (81.8 %, Fig. 2a). Similarly, the percentage of benzodiazepine users at weeks 12 and 24 was significantly lower in the mirtazapine group (11.1 and 12.5 %) than it was in the SSRIs group (85.7 and 81.8 %, Fig. 2b, c), respectively. Comparing the three antidepressant groups, the percentage of benzodiazepine users in the mirtazapine group was significantly lower than that sertraline and paroxetine groups were at weeks 6, 12, and 24 (Fig. 3a–c). Conversely, there were no significant differences in the percentages of benzodiazepine users between the mirtazapine and SSRIs groups at weeks 1 and 2 (52.9 vs. 72.1 and 53.3 vs. 66.7 %, respectively).

**Table 3:** Frequencies of benzodiazepine use in participants

	Baseline^a^	Week 1	Week 2	Week 6	Week 12	Week 24
**Mirtazapine**	***n*** **=** **20**	***n*** **=** **17**	***n*** **=** **15**	***n*** **=** **14**	***n*** **=** **9**	***n*** **=** **8**
Non-use, *n* (%)		8 (47.1)	7 (46.7)	11 (78.6)	8 (88.9)	7 (87.5)
1–6 days per week, *n* (%)		5 (29.4)	4 (26.7)	1 (7.1)	0 (0.0)	0 (0.0)
Every day, *n* (%)		4 (23.5)	4 (26.7)	2 (14.3)	1 (11.1)	1 (12.5)
**SSRIs**	***n*** **=** **45**	***n*** **=** **43**	***n*** **=** **42**	***n*** **=** **33**	***n*** **=** **28**	***n*** **=** **22**
Non-use, *n* (%)		12 (27.9)	14 (33.3)	6 (18.2)	4 (14.3)	4 (18.2)
1–6 days per week, *n* (%)		15 (34.9)	8 (19.0)	12 (36.4)	9 (32.1)	4 (18.2)
Every day, *n* (%)		16 (37.2)	20 (47.6)	15 (45.5)	15 (53.6)	14 (63.6)
**Sertraline**	***n*** **=** **28**	***n*** **=** **26**	***n*** **=** **26**	***n*** **=** **22**	***n*** **=** **19**	***n*** **=** **15**
Non-use, *n* (%)		7 (26.9)	9 (34.6)	5 (22.7)	3 (15.8)	3 (20.0)
1–6 days per week, *n* (%)		8 (30.8)	4 (15.4)	7 (31.8)	5 (26.3)	2 (13.3)
Every day, *n* (%)		11 (42.3)	13 (50.0)	10 (45.5)	11 (57.9)	10 (66.7)
**Paroxetine**	***n*** **=** **17**	***n*** **=** **17**	***n*** **=** **16**	***n*** **=** **11**	***n*** **=** **9**	***n*** **=** **7**
Non-use, *n* (%)		5 (29.4)	5 (31.3)	1 (9.1)	1 (11.1)	1 (14.3)
1–6 day per week, n (%)		7 (41.2)	4 (25.0)	5 (45.5)	4 (44.4)	2 (28.6)
Every day, *n* (%)		5 (29.4)	7 (43.8)	5 (45.5)	4 (44.4)	4 (57.1)

*SSRI* selective serotonin reuptake inhibitor

^a^Patients prescribed benzodiazepines at baseline (day 1) were only counted in numbers in this table

### Efficacy

Regardless of whether the participants received benzodiazepine prescriptions from day 1, the average HDRS, SDS, AIS, and CGI-S total scores for each group were significantly decreased compared with those at the baseline, as determined using the MMRM (*P* < 0.05). Table 4 shows the sequential measurements of the efficacy outcomes for all participants. The difference in the changes in the HDRS scores were not statistically significant between the mirtazapine and SSRIs groups (*F* = 0.37, *df* = 1, 78; mean difference, 95 % confidence interval [CI] −0.78 [–3.31 to 1.76], *P* = 0.54) or among the three groups (*F* = 0.49, *df* = 2, 76, *P* = 0.62). In addition, there was no statistical difference in the changes in the AIS and CGI-S scores between the mirtazapine and SSRIs groups (AIS: *F* = 2.23, *df* = 1, 73; mean difference, 95 % CI −1.32 [–3.07 to 0.44], *P* = 0.14; CGI-S: *F* = 1.11, *df* = 1, 78; mean difference, 95 % CI −0.19 [–0.56 to 0.17], *P* = 0.30), and among the three groups (AIS: *F* = 3.10, *df* = 2, 70, *P* = 0.051; CGI-S: *F* = 0.80, *df* = 2, 76, *P* = 0.45). Regarding the SDS, the difference in the changes in SDS scores was not statistically significant between the mirtazapine and SSRIs groups (*F* = 3.40, *df* = 1, 79; mean difference, 95 % CI −3.30 [−6.86 to 0.26], *P* = 0.069); however, there was a significant difference among the three groups (*F* = 3.29, *df* = 2, 76, *P* = 0.043). Specifically, there were significantly different changes in the SDS scores between the mirtazapine and paroxetine groups (mean difference 95 % CI −5.74 [−11.22 to −0.25], *P* = 0.038), indicating that the SDS scores of the mirtazapine group had improved more significantly than those of the paroxetine group. Similarly, the analyses of the data of patients who were prescribed benzodiazepines from day 1 revealed that the differences in the changes from the baseline HDRS, AIS, and CGI-S scores were not statistically significant between the mirtazapine and SSRIs groups as well as among the three groups (data not shown). In contrast to the analysis of the data of all the participants, there were no significant differences in the HDRS, AIS, and CGI-S (data not shown) as well as the SDS between the mirtazapine and SSRIs groups (SDS: *F* = 3.05, *df* = 1, 67, *P* = 0.085) and among the three groups (SDS: *F* = 2.45, *df* = 2, 64, *P* = 0.095) in the patients who were prescribed benzodiazepines from day 1.

**Table 4:** Sequential measurements of clinical efficacy outcomes

Variables	Baseline	Week 1	Week 2	Week 6	Week 12	Week 24
	Estimated marginal means (SE)					
HDRS						
Mirtazapine	23.0 (1.2)	19.0 (1.3)	15.5 (1.3)	9.6 (1.4)	9.3 (1.5)	5.9 (1.6)
SSRIs	23.1 (0.9)	19.2 (0.9)	16.9 (0.9)	12.9 (1.0)	9.5 (1.0)	5.4 (1.1)
Sertraline	23.2 (1.1)	19.1 (1.2)	16.3 (1.1)	12.1 (1.2)	8.7 (1.3)	5.3 (1.4)
Paroxetine	22.9 (1.5)	19.4 (1.5)	17.8 (1.5)	14.6 (1.7)	11.2 (1.8)	5.8 (2.0)
SDS						
Mirtazapine	56.0 (1.7)	52.4 (1.8)	46.6 (1.8)	44.0 (1.9)	43.7 (2.1)	38.0 (2.2)
SSRIs	57.6 (1.3)	54.8 (1.3)	52.8 (1.3)	48.7 (1.4)	44.9 (1.5)	41.8 (1.6)
Sertraline	57.4 (1.6)	54.1 (1.7)	51.2 (1.6)	47.2 (1.7)	42.4 (1.8)	40.3 (1.9)
Paroxetine	57.9 (2.1)	56.1 (2.1)	55.0 (2.1)	51.4 (2.3)	49.8 (2.5)	44.5 (2.8)
AIS						
Mirtazapine	11.5 (0.9)	8.4 (0.9)	6.6 (0.9)	5.8 (1.0)	6.8 (1.1)	5.0 (1.1)
SSRIs	12.9 (0.6)	10.2 (0.6)	9.3 (0.6)	7.5 (0.7)	6.6 (0.7)	5.5 (0.8)
Sertraline	12.8 (0.8)	10.0 (0.8)	8.1 (0.8)	6.9 (0.8)	5.3 (0.9)	4.6 (1.0)
Paroxetine	13.1 (1.0)	10.5 (1.0)	11.3 (1.0)	8.8 (1.2)	9.2 (1.2)	7.2 (1.4)
CGI-S						
Mirtazapine	4.4 (0.2)	3.8 (0.2)	3.2 (0.2)	2.8 (0.2)	2.6 (0.2)	1.8 (0.2)
SSRIs	4.3 (0.1)	4.0 (0.1)	3.6 (0.1)	3.1 (0.1)	2.7 (0.1)	2.1 (0.2)
Sertraline	4.4 (0.2)	4.0 (0.2)	3.6 (0.2)	3.0 (0.2)	2.6 (0.2)	2.1 (0.2)
Paroxetine	4.2 (0.2)	4.1 (0.2)	3.6 (0.2)	3.5 (0.2)	3.0 (0.3)	2.0 (0.3)

All values are based on estimated marginal means using a linear mixed effects model for repeated measures data

*SSRI* selective serotonin reuptake inhibitor, *HDRS* 17-item Hamilton depression rating scale, *SDS* Zung self-rating depression scale, *AIS* Athens insomnia scale, *SE* standard error

### Safety analysis

Table 5 shows the details of all treatment-emergent AEs observed in this study. The AEs that led to the discontinuation of study participation appeared within the first 2 weeks except for the case of abnormal liver function tests, which was observed at week 6. However, the affected patients recovered after withdrawing from the study except for the patients with the SAEs. The analysis of the incidence rate of AEs revealed that the proportions of the patients with any AEs differed among the three antidepressant groups (*χ* ^2^ = 12.5, *df* = 2, *P* = 0.0019). Specifically, the percentage of patients with any AEs was significantly lower in the sertraline (7/32, 21.9 %) group than in the mirtazapine (16/27, 59.3 %, *χ* ^2^ = 8.6, *df* = 1, *P* = 0.034) and paroxetine (12/18, 66.7 %, *χ* ^2^ = 9.8, *df* = 1, *P* = 0.0017) groups.

**Table 5:** Summary of treatment-emergent adverse events (AEs)

	Mirtazapine, *n* = 27	Sertraline, *n* = 32	Paroxetine, *n* = 18
	*n* (%)	*n* (%)	*n* (%)
Total number of patients with AEs	16 (59.3)	7 (21.9)	12 (66.7)
Serious AEs (SAEs)	0 (0.0)	2 (6.3)	0 (0.0)
Brain hemorrhage^a^	0 (0.0)	1 (3.1)	0 (0.0)
Hospitalization due to depression deterioration	0 (0.0)	1 (3.1)	0 (0.0)
AEs leading to discontinuation except for SAEs	7 (25.9)	1 (3.1)	5 (27.8)
Sedation including somnolence	3 (11.1)	0 (0.0)	0 (0.0)
Insomnia	1 (3.7)	0 (0.0)	0 (0.0)
Abnormal liver function test	1 (3.7)	0 (0.0)	0 (0.0)
Eruption	1 (3.7)	0 (0.0)	0 (0.0)
Dysgeusia	1 (3.7)	0 (0.0)	0 (0.0)
Nausea	0 (0.0)	1 (3.1)	1 (5.6)
Sexual dysfunction (erection failure)	0 (0.0)	0 (0.0)	1 (5.6)
Mania	0 (0.0)	0 (0.0)	2 (11.1)
Panic attack	0 (0.0)	0 (0.0)	1 (5.6)
Specific symptoms of AEs except for SAEs			
Sedation including somnolence	9 (33.3)	0 (0.0)	3 (16.7)
Insomnia	2 (3.7)	1 (3.1)	0 (0.0)
Akathisia	1 (3.7)	0 (0.0)	2 (11.1)
Irritability	1 (3.7)	1 (3.1)	1 (5.6)
Mania	0 (0.0)	0 (0.0)	2 (11.1)
Weight increased	3 (11.1)	0 (0.0)	0 (0.0)
Increased appetite	1 (3.7)	0 (0.0)	0 (0.0)
Headache	0 (0.0)	0 (0.0)	2 (11.1)
Dizziness	0 (0.0)	0 (0.0)	1 (5.6)
Nausea	1 (3.7)	5 (15.6)	4 (22.2)
Fatigue	3 (11.1)	0 (0.0)	2 (11.1)
Eruption	1 (3.7)	0 (0.0)	0 (0.0)
Abnormal liver function test	1 (3.7)	0 (0.0)	0 (0.0)
Dysgeusia	1 (3.7)	0 (0.0)	0 (0.0)
Sexual dysfunction (erection failure)	0 (0.0)	0 (0.0)	1 (5.6)
Hyperhidrosis	0 (0.0)	0 (0.0)	2 (11.1)
Constipation	0 (0.0)	0 (0.0)	1 (5.6)

*AE* adverse event, *SAE* serious adverse event

^a^Brain hemorrhage was unrelated to sertraline administration according to the diagnosis by the neurosurgeon. All AEs were treatment emergent

### Relationship between clinical responses and serum BDNF levels

Table 6 shows the comparisons of the baseline levels of mature BDNF, proBDNF, and their ratios between the responders and non-responders in both groups at week 6. There were no significant differences in the baseline levels of each BDNF protein between the two groups (Table 6).

**Table 6:** Baseline serum brain-derived neurotrophic factor (BDNF) levels of responders and non-responders at week 6

	Responders, *n* = 24	Non-responders, *n* = 29	Statistics	*P*
Levels at baseline				
Mature BDNF (ng/mL), mean (SD)	12.8 (3.8)	13.4 (3.4)	*t* = −0.67, *df* = 51	0.51
ProBDNF (pg/mL), median [quartiles]	607.5 [84.4, 5158.3]	135.0 [45.6, 2803.5]	*Z* = −1.3	0.18
Ratio of mature BDNF/proBDNF^a^	25.7 [2.3, 146.8]	105.7 [4.7, 309.2]	*Z* = −1.3	0.21

Responders and non-responders were assessed at week 6

*BDNF* brain-derived neurotrophic factor, *SD* standard deviation

^a^Ratio is serum level of mature BDNF (pg/mL) divided by that of proBDNF (pg/mL) in each individual. Serum mature BDNF levels were analyzed using Student *t* test. Serum proBDNF and ratio of mature BDNF/proBDNF were analyzed using Mann–Whitney *U* test

Table 7 shows the long-term effectiveness of the antidepressant treatments on serum BDNF levels in 27 responders of both groups on the final assessment day at week 24. Of the 35 patients who completed the study, there were technical failures in the samples of five while three did not achieve a clinical response by week 24. The serum levels of the mature BDNF decreased significantly between weeks 6 and 12 from the baseline levels but the change did not persist (Table 7). Furthermore, the serum proBDNF levels of the responders who achieved clinical responses by week 24 were statistically significantly decreased when compared to the baseline levels (Table 7).

**Table 7:** Long-term changes in serum levels of brain-derived neurotrophic factor (BDNF) in responders at week 24

	Baseline	Week 6	Week 12	Week 24	Statistics	*P*
Mature BDNF (ng/mL), EMS (SE)	12.7 (0.7)	11.2 (0.7)^a^	11.8 (0.7)^a^	12.1 (0.7)	*F* = 3.5, *df* = 2.4	0.027*
ProBDNF (pg/mL), median [quartiles]	634.7 [92.4, 5381.8]	507.9 [95.6, 4975.8]	484.5 [82.5, 4471.0]	463.5 [109.5, 4018.4]^b^	*χ* ^2^ = 8.5, *df* = 3	0.036*
Ratio of mature BDNF/proBDNF, median [quartiles]	22.7 [2.1, 135.6]	27.0 [2.3, 115.5]	29.3 [2.7, 127.8]	30.4 [3.1, 153.6]	*χ* ^2^ = 1.6, *df* = 3	0.67

Serum mature BDNF levels were analyzed using repeated measure analysis of variance (ANOVA). Adjustment for multiple comparisons was Bonferroni. Serum proBDNF levels and ratio of mature BDNF/proBDNF levels were analyzed using Friedman’s test followed by Wilcoxon signed rank test

*BDNF* brain-derived neurotrophic factor, *EMS* estimated marginal means, *SE* standard error, *CI* confidence interval

^a^Mean differences in serum mature BDNF levels at week 6 (−1.4 ng/mL, SE = 0.5, 95 % CI −2.7 to –0.07, *P* = 0.035) and at week 12 (−0.8 ng/mL, SE = 0.3, 95 % CI −1.7 to −0.01, *P* = 0.045) were significantly decreased compared to the baseline levels

^b^Serum proBDNF levels at week 24 were significantly decreased compared to the baseline levels (*Z* = −2.4, *P* = 0.019). *P < 0.05, *n* = 27

## Discussion

Three interesting results in this study are of particular significance and worth expounding. First, among the patients with depression who were prescribed both an antidepressant and benzodiazepines from the beginning of the treatment, our results showed that there was a significantly smaller proportion of benzodiazepine users in the mirtazapine treatment group than there was in the SSRIs treatment group. However, the efficacy of mirtazapine in treating depression was not different from that of the SSRIs. Second, the safety assessment revealed that the proportion of patients who experienced treatment-emergent AEs was significantly lower in the sertraline group than it was in the mirtazapine and paroxetine groups. Third, the present study showed that the serum proBDNF levels of the responders who achieved clinical responses in both antidepressant groups at the final assessment day, at week 24, were significantly decreased compared to the baseline levels, while the serum mature BDNF levels significantly decreased from week 6 to 12, but only temporarily, and this effect did not persist till week 24.

The results of our analysis revealed that among the depressed patients prescribed both an antidepressant and benzodiazepines at the beginning of treatment, there was a significantly smaller proportion of benzodiazepine users that were treated with mirtazapine than were treated with SSRIs. However, the efficacy of mirtazapine in depression treatment was not different from that of the SSRIs. These results are compatible with our hypothesis. A previous meta-analysis of the discontinuation of benzodiazepine use demonstrated that the effective strategies are mainly psychological interventions combined with regimens such as a gradual reduction in the dose of prescribed benzodiazepines [26–28]. Although numerous studies have indicated the benefits of discontinuing benzodiazepine use in pharmacotherapy, effective pharmacological interventions have not yet been established to replace them [26–29]. Although restricting or discontinuing the use of benzodiazepines is strongly recommended in the treatment of depression, this has been challenging to achieve in routine clinical practice [26]. Therefore, antidepressant treatments without benzodiazepines from the acute phase or the first stage of treatment of major depression are considered useful for reducing the number of benzodiazepine users. Furthermore, the findings of the present study have identified the antidepressant from the first-line recommended agents that influence the persistent use of benzodiazepines in the treatment of patients with MDD. Specifically, our results suggest that prescribing mirtazapine as the first antidepressant to be administered could potentially prevent patients who are depressed from having to continuously take benzodiazepines. Further comprehensive, double-blind studies would be required to confirm this finding.

The efficacy analysis of this study revealed there were no statistically significant differences in the changes in the HDRS scores between the mirtazapine and the SSRIs groups as well as between the three groups. These results are consistent with the findings of a meta-analysis study of mirtazapine versus other antidepressants including SSRIs [13]. Additionally, the mirtazapine group improved more than the paroxetine group did in the change in SDS scores. It is difficult to explain the discrepancy between the HDRS and SDS scores of the mirtazapine and paroxetine groups in this study because two meta-analysis studies previously demonstrated a lack of difference in the efficacy of mirtazapine and paroxetine [13, 30]. A plausible explanation is that the paroxetine group had a smaller size than the mirtazapine group did, which might have influenced the results. The efficacy of ameliorating sleep disturbances, as determined by the AIS assessment, showed no statistically significant differences between the groups. Considering that the efficacy of mirtazapine in treating depressive symptoms and sleep disturbances is not different from that of the SSRIs, the present findings could support mirtazapine as the first choice for the treatment of major depression because of its advantage of decreasing the benzodiazepine requirement compared to the SSRIs.

The safety analysis demonstrated that the proportion of patients who experienced treatment-emergent AEs was significantly lower in the sertraline group than it was in the mirtazapine and paroxetine groups. These results are consistent with the findings of a previous meta-analysis study [31] that demonstrated the high tolerability of sertraline and relatively low tolerability of mirtazapine and paroxetine in patients with MDD.

Focusing on the AEs of mirtazapine, our results showed that sedation, including somnolence, very likely caused the discontinuation of the drug in the early stage of the treatment of major depression. Although it has been reported that the effectiveness of mirtazapine on sleep disturbance appears very quickly [32], sedation caused by mirtazapine occurs with high frequency (50 % or more) [9]. The improvement of sleep disturbance and sedation with mirtazapine treatment is thought to be inextricably linked. Therefore, mirtazapine as the first-choice agent in depression treatment could be expected to effectively treat depression without the use of benzodiazepines by its rapid onset of clinical action and improvement of sleep disturbance [12, 13, 32, 33]. However, it would be necessary to implement considerations and strategies to reduce the risk of early dropout due to sedation.

The present study showed that serum proBDNF levels of the responders who achieved clinical responses in both antidepressant groups at the final assessment day were significantly decreased at week 24 compared to the baseline levels. Furthermore, the serum mature BDNF levels significantly decreased from week 6 to 12, but the change did not persist up to week 24. To the best of our knowledge, this is the first report to show the changes in serum levels of mature BDNF and proBDNF following antidepressant treatment in patients who are depressed and achieved clinical responses. A previous study by Yoshimura et al. [16] reported there were no changes in the serum levels of mature BDNF and proBDNF in patients with MDD, who were administrated fluvoxamine for 4 weeks. Our findings are inconsistent with their results, and a plausible reason is that the experimental conditions of these two studies differed. Specifically, our present study focused on clinical responders, and the duration was 24 weeks, which differed from that of Yoshimura et al. [16] that had a 4-week duration. The present results may not have provided practical biomarkers as predictors of clinical responses because the serum levels of mature BDNF changed erratically and the decrease in serum proBDNF levels was too slow. However, our present findings may contribute to the understanding of the physiological roles of mature BDNF and proBDNF in the timing of the clinical responses and effectiveness of antidepressant treatments in patients with MDD. The physiological mechanisms and dynamics of serum mature BDNF and proBDNF levels in mood disorders such as major depression and bipolar disorder are still unclear and remain to be elucidated. Furthermore, a recent meta-analysis reports that the peripheral blood levels of BDNF in patients with bipolar disorder with manic and depressive episodes are decreased, but those with euthymia are not altered compared to healthy controls [34]. In contrast, Södersten et al. [17] reported that the serum levels of mature BDNF are higher in patients with bipolar disorder than they are in the controls. Further studies are needed to identify the effects of antidepressants on blood levels of mature BDNF and proBDNF using larger sample sizes, to clarify their physiological mechanisms in mood disorders such as major depression and bipolar disorder.

In addition, there were no differences in the level of mature BDNF, proBDNF, and the ratio of mature BDNF/proBDNF at the baseline between the responders and non-responders assessed at week 6. Previous studies, which did not distinguish between mature BDNF and proBDNF, showed incongruous findings that serum BDNF levels would be useful as a predictor of responses to antidepressant treatments in patients who are depressed [35–37]. Our results do not support measuring mature BDNF and proBDNF at pre-treatment as a useful predictor of responses to antidepressant treatment in patients with MDD.

There are four main limitations to this study, which are worth mentioning. First, as a prospective, randomized, open-label, blinded endpoint (PROBE) procedure, the investigators were aware of the primary endpoint in this study. Therefore, there was a possibility that the investigators emphasized to the patients the effect of mirtazapine on sleep disturbance. This could have led to a potential placebo effect on those who took mirtazapine. Furthermore, this issue is a technical inevitability in an open study. Therefore, a double-blind, randomized clinical trial (RCT) would be needed to confirm our results. Second, the numbers of dropouts were too numerous to accurately assess the effects of antidepressant treatments in this study. Regarding the pragmatic aspects of conducting clinical trials, Rutherford et al. [38] reported that the frequency of patient visits influences the dropout rate in antidepressant treatment [38]. The assessment intervals in this study were 1 week or more even in the first 4 weeks because the priority was to ensure a routine psychiatric practice setting was maintained above the experimental considerations. Previous survey studies of antidepressant prescriptions for treating depression in general clinical practice demonstrated that patients discontinue an initial antidepressant in the first 4 weeks at a rate of 26.2–42.4 % [39–42]. The present results of the dropout rate evaluation were similar to the previously reported rates in general clinical practice [39–42]. To elucidate the effectiveness of antidepressants on the continuous use of benzodiazepines further, the rate of visit frequencies of future studies should be higher than they were in this study. Third, sertraline and paroxetine were not randomized in this study. The patients randomly assigned to the SSRIs group were prescribed sertraline or paroxetine according to each investigator’s assessment and judgment. We incorporated a pragmatic trial design into real-life practice settings rather than an exploratory study design [43]. To clarify the findings of the present study, further studies that are strictly designed, such as a double-blind RCT, are necessary. Fourth, the sample size of this study was small and, therefore, we were unable to examine the potential applicability of the serum BDNF level as a biomarker of clinical antidepressant drug responses. Further studies with a larger sample size would be required to verify this.

## Conclusions

This study showed the possibility of mirtazapine as the first-choice antidepressant for current depressive episodes by revealing its potential as an effective strategy to reduce the use of benzodiazepines in patients with major depression.

## Abbreviations

SSRI: selective serotonin reuptake inhibitor
MDD: major depressive disorder
BDNF: brain-derived neurotrophic factor
HDRS: Hamilton depression rating scale
TrkB: tropomyosin receptor kinase B
UMIN: University Hospital Medical Information Network
ELISA: enzyme-linked immunosorbent assay
SDS: Zung self-rating depression scale
AIS: Athens insomnia scale
CGI-S: global impressions-severity
AE: adverse event
MMRM: mixed effects model for repeated measures
ANOVA: repeated analysis of variance
SPSS: statistical package for the social sciences
CR: controlled release
SAE: serious adverse event
PROBE: prospective, randomized, open-label, blinded endpoint
RCT: randomized clinical trial
